# The Biological Fate of a Novel Anticancer Drug Candidate TNBG-5602: Metabolic Profile, Interaction with CYP450, and Pharmacokinetics in Rats

**DOI:** 10.3390/molecules27082594

**Published:** 2022-04-18

**Authors:** Rui Li, Sha Zhou, Zongjie Gan, Lijuan Wang, Yu Yu

**Affiliations:** 1 College of Pharmacy, Chongqing Medical University, Chongqing 400016, China; 2016010166@stu.cqmu.edu.cn (R.L.); gzj@cqmu.edu.cn (Z.G.); 2 Yaopharma Co., Ltd., Chongqing 401121, China; zhousha@yaopharma.com; 3 Department of Pharmaceutics, Chongqing Engineering Research Center of Pharmaceutical Sciences, Chongqing Medical and Pharmaceutical College, Chongqing 401331, China; 10731@cqmpc.edu.cn

**Keywords:** TNBG-5602, metabolism, biotransformation pathway, molecular docking, pharmacokinetics, IC_50_, drug–enzyme interaction

## Abstract

TNBG-5602, a novel anticancer drug candidate, may induce the expression of PPARγ, causing targeted lipotoxicity in cancer tissues. In this study, the in vivo metabolism in rats, in vitro metabolism in recombinant cytochromes, molecular docking for the CYP binding site, and pharmacokinetics in rats were explored to better understand TNBG-5602′s in vivo fate and behavior. Thirteen metabolites were identified using a high-resolution mass spectrometry method, and metabolizing pathways of TNBG-5602 were proposed. Results suggest that TNBG-5602 could be metabolized by CYP450s, while CYP2D6 may play an important role in its in vivo metabolism. The main metabolizing sites of TNBG-5602 are the amino group on the side chain and rings A and E in the molecule. TNBG-5602 is a potent CYP2D6 inhibitor, with an IC_50_ value of 2.52 μM. An interaction responsible for its metabolism is formed by the NH on the side chain bonding with the ASP301 on the CYP2D6. The pharmacokinetics in rats after a single intravenous administration were fitted to a two-compartment model. The clearance was 0.022 L min^−1^, and the elimination half-life was 710.9 min. The distribution volume of the peripheral compartment was 1.88-fold that of the central compartment, while the K_12_ was 1.5-fold that of K_21_. In conclusion, these studies have not only revealed the metabolizing pathways of TNBG-5602 using in vivo and in vitro methodology, but they have also provided the pharmacokinetic characteristics of TNBG-5602 in rats. The results suggest that TNBG-5602 has good drug developability in terms of pharmacokinetic behaviors.

## 1. Introduction

The economic burden of cancer diseases is rapidly growing worldwide, and cancer could be the top leading cause of death in 2019, as suggested by the World Health Organization (WHO) [1]. It is anticipated that the numbers of new cancer cases and cancer deaths in 2020 may reach 19.3 and 10.0 million [2], respectively. Moreover, new cancer cases are projected to rise to 28.4 million by 2040 [2]. Despite the significant progress made in chemotherapy in recent decades, the treatment is still constrained by many problems such as drug resistance and severe toxicity [3,4]. Hence, novel anticancer drugs with improved efficacy and fewer side effects are urgently needed to satisfy unmet clinical needs.

**Figure 1:**
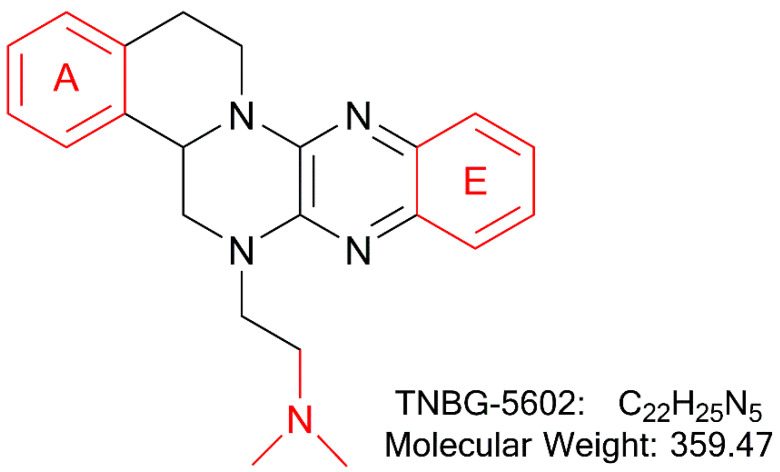
The chemical structure of TNBG-5602, a new isoquinoline derivative with a sterol structure, that showed strong inhibition against the proliferation of cancer cell lines by interfering with lipid metabolism. The main metabolic sites of TNBG-5602 were the rigid coplanar ring (ring A and ring E) in its structure and the side chain.

In recent years, studies suggest that interfering with lipid metabolism anticipates being a promising targeted anticancer strategy [5,6,7,8]. As previously reported, TNBG-5602 (Figure 1), a new isoquinoline derivative with a sterol structure, showed strong inhibitory activity against five human cancer cell lines [9]. Lipid (oil red O) staining illustrated that TNBG-5602 could selectively cause lipid accumulation in cancer cells and tissues [10]. Thus, the pharmacological action of TNBG-5602 could be due to interference with the lipid metabolism in cancer cells. Our preliminary studies also found that TNBG-5602 effectively inhibited the proliferation of targeted liver cancer cells in vitro with low toxicity to normal tissues via upregulating peroxisome proliferator-activated receptor γ (PPARγ) [10]. Additionally, using gene chips and proteomics, it has been demonstrated that TNBG-5602 may have an effect by upregulating phosphatase and tensin homolog deleted on chromosome 10 (PTEN), thereby blocking the phosphorylation of Akt [11]. Thus, TNBG-5602 could be a promising new anticancer drug candidate. However, the pharmacokinetic properties of TNBG-5602 have not been reported so far. Understanding its pharmaceutics is important to determine the drug developability of the compound.

On the other hand, many studies have shown that several anticancer drugs, such as selective inducers or inhibitors of CYPs, can regulate the activity and alter the expression of CYPs to cause drug-drug interaction or even hepatotoxicity [12,13,14,15]. Therefore, it is also very important to understand how the absorption and metabolism of an anticancer drug will alter molecular mechanisms underlying potential drug-drug interactions and to support the design of a new drug candidate. Any effect of TNBG-5602 on CYPs also remains unknown. 

To support the clinical development of TNBG-5602, studies were performed with the goal to reveal how TNBG-5602 interacts with drug metabolizing enzymes, its biological fate, and its pharmacokinetic properties. Results from the studies will provide meaningful information as a scientific basis for further clinical investigations on TNBG-5602.

## 2. Results

### 2.1. Identification of Structure of TNBG-5602′s Metabolites

**Figure 2:**
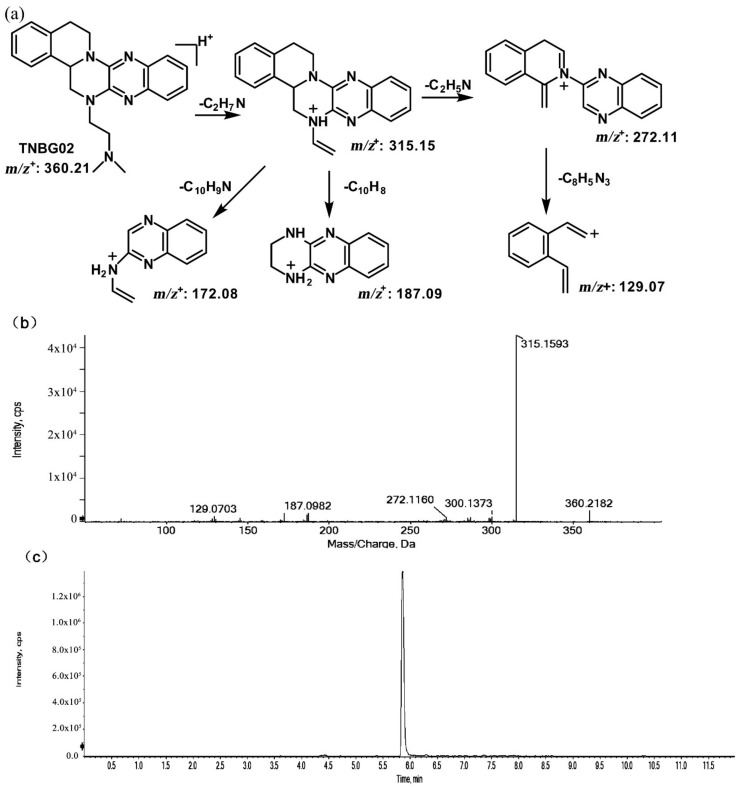
Suggested MS/MS fragmentation patterns of TNBG-5602 (**a**), the representative MS/MS spectra of TNBG-5602 with an [M + H]^+^ ion at *m*/*z* 360.21 with major fragmentation ions at *m*/*z* values 315.15, 272.11, and 172.08 (**b**), and the representative chromatogram of TNBG-5602 to elute at a retention time of 5.8 min when separated on the UPLC-Q-TOF-MS/MS (**c**).

The structures of thirteen TNBG-5602 metabolites, following in vivo study in rats, were successfully identified using an LC-MS/MS method, including *m*/*z* 376.21, *m*/*z* 346.20, *m*/*z* 374.19, *m*/*z* 390.19, *m*/*z* 392.20, and *m*/*z* 332.18. Chemical structures of the main metabolites were elucidated, and a metabolizing pathway of TNBG-5602 in vivo was proposed. High-resolution LC-MS/MS showed TNBG-5602 to elute at a retention time of 5.8 min with an [M + H]^+^ ion at *m*/*z* 360.21 with major fragmentation ions at *m*/*z* values 315.15, 272.11, and 172.08. The fragmentation patterns and representative spectra of TNBG-5602 are shown in Figure 2. Each of these ions is structurally informative with respect to the corresponding fragmentation ions produced, and they are described below for each of the metabolites represented by M1–M13. The metabolites, along with their LC-MS/MS retention times and related *m*/*z* values, and the correspondence between the theoretical and actual mass values measured are summarized in Table 1.

#### 2.1.1. Metabolites of *m*/*z* 376.21

**Figure 3:**
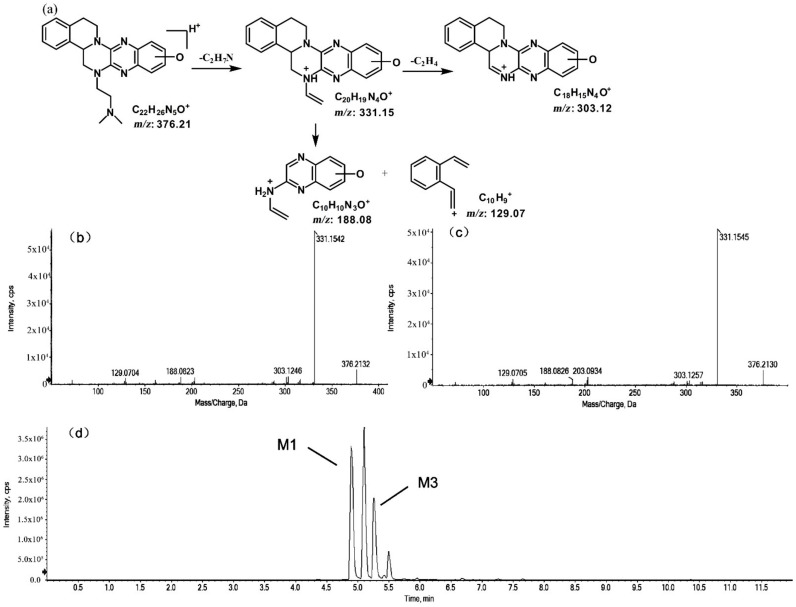
Suggested MS/MS fragmentation patterns of the pair of structural isomers (M1 and M3) identified to be C_22_H_25_N_5_O with *m*/*z* 376.21 (**a**), MS/MS spectra of M1 eluted at a retention time of 4.9 min (**b**), MS/MS spectra of M3 eluted at a retention time of 5.3 min (**c**), and the representative chromatogram of M1 and M3 when separated on the UPLC-Q-TOF-MS/MS (**d**).

**Figure 4:**
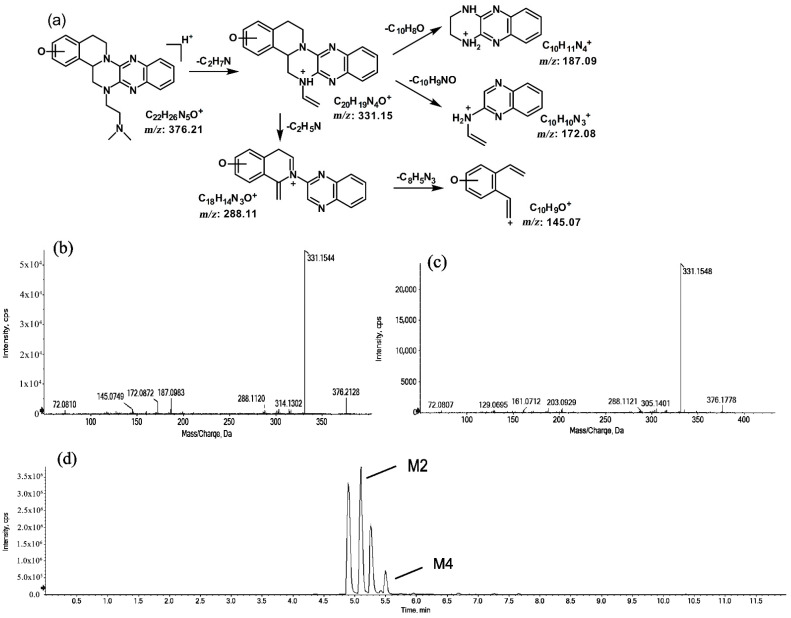
Suggested MS/MS fragmentation patterns of the pair of structural isomers (M2 and M4) identified to be C_22_H_25_N_5_O with *m*/*z* 376.21 (**a**), MS/MS spectra of M2 eluted at a retention time of 5.1 min (**b**), MS/MS spectra of M4 eluted at a retention time of 5.5 min (**c**), and the representative chromatogram of M2 and M4 when separated on the UPLC-Q-TOF-MS/MS (**d**).

The metabolites of *m*/*z* 376.21 were eluted at retention times of 4.9, 5.1, 5.3, and 5.5 min. These metabolites (M1, M2, M3, and M4) were structural isomers with the same precursor ion at *m*/*z* 376.21 and characteristic MS/MS product ions at *m*/*z* 331.15. These molecular ions and their characteristic product ions were all 16 Da greater than the prototype at *m*/*z* 360.21 and its product ion at *m*/*z* 315.15. These results indicate that M1–M4 should be considered as the mono-oxidation product of the prototype. Furthermore, the different product ions provided convincing evidence of their different oxidation sites. M1 and M3, which featured the same product ions of *m/z* 188.08 and *m*/*z* 129.07, should be a pair of isomers oxidized on ring E. However, information about the specific oxidation sites of M1 and M3 on ring E could not be obtained from their MS/MS profiles. The fragmentation patterns, representative spectra, and chromatograms of M1 and M3 are shown in Figure 3. M2 and M4, with the product ions *m*/*z* 172.08 and *m*/*z* 145.07, were a pair of isomers oxidized on ring A. Similarly, information about their specific oxidation site could not be obtained from their MS/MS profiles. The proposed fragmentation patterns, representative spectra, and chromatograms of M2 and M4 are shown in Figure 4.

#### 2.1.2. Metabolites of *m*/*z* 346.20

**Figure 5:**
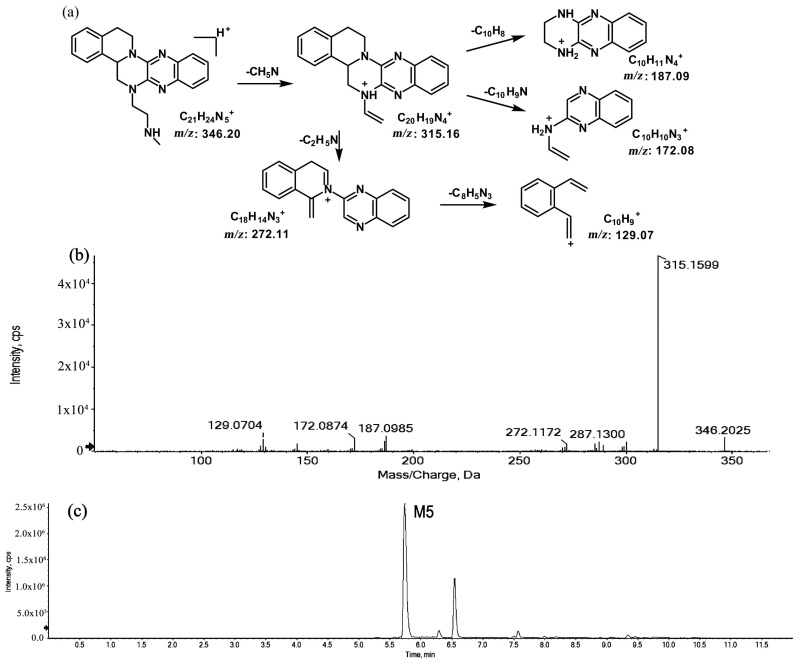
Suggested MS/MS fragmentation patterns of M5 identified to be C_21_H_23_N_5_ with *m*/*z* 346.20 (**a**), MS/MS spectra of M5 eluted at a retention time of 5.7 min (**b**), and the representative chromatogram of M5 when separated on the UPLC-Q-TOF-MS/MS (**c**).

**Figure 6:**
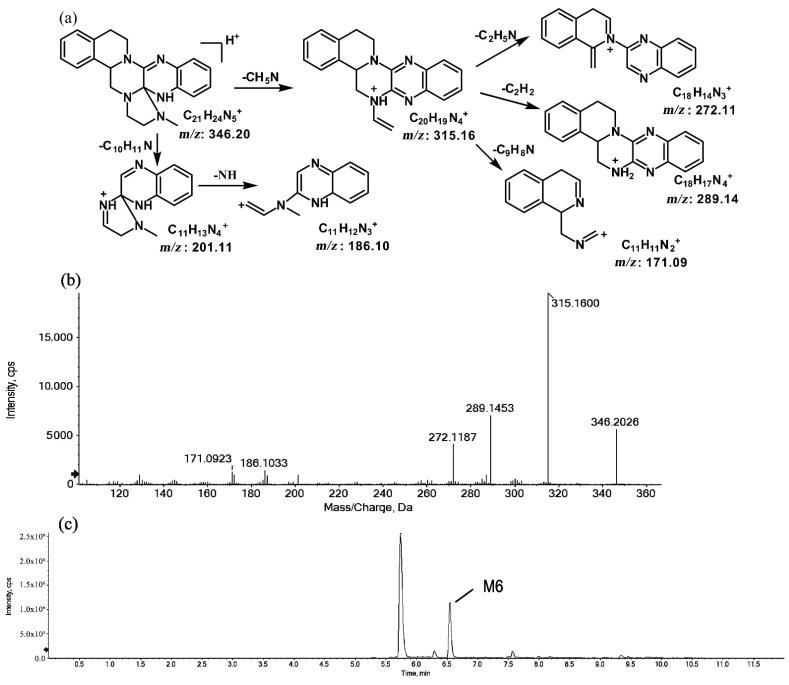
Suggested MS/MS fragmentation patterns of M6 identified to be C_21_H_23_N_5_ with *m*/*z* 346.20 (**a**), MS/MS spectra of M6 eluted at a retention time of 6.5 min (**b**), and the representative chromatogram of M6 when separated on the UPLC-Q-TOF-MS/MS (**c**).

The metabolites of *m*/*z* 346.20 (M5 and M6) were found to be structural isomers with the same precursor ions at *m*/*z* 346.20, which decreased by 14 Da compared to those of the prototype, indicating that the fragmentation pathway could be the demethylation of the prototype. The retention times of M5 and M6 were 5.7 and 6.5 min, respectively. M5 yielded product ions at *m*/*z* 315.15, 272.11, and 172.08. The proposed fragmentation patterns of M5 and the representative spectra are shown in Figure 5. The product ions of M6 at *m*/*z* 289.14, 201.11, and 171.09 were different from those of M5. It can be postulated that M5 was formed via N-demethylation on the side chain at the initiation Then, the amino group on the M5 side chain formed a ring with the core structure to form M6. The proposed fragmentation patterns are shown in Figure 6.

#### 2.1.3. Metabolites of *m*/*z* 374.19

**Figure 7:**
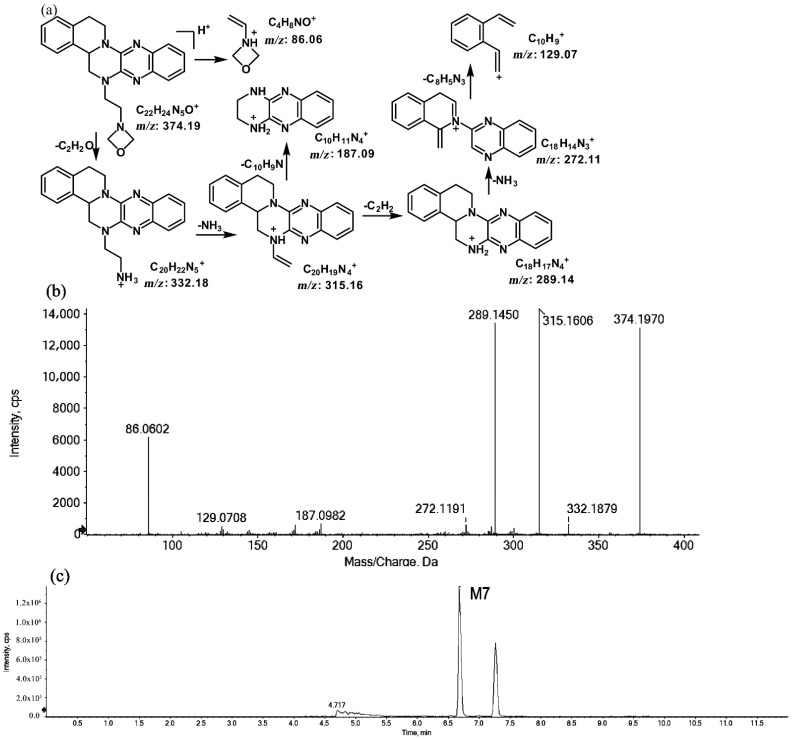
Suggested MS/MS fragmentation patterns of M7 identified to be C_22_H_23_N_5_O with *m*/*z* 374.19 (**a**), MS/MS spectra of M7 eluted at a retention time of 6.6 min (**b**), and the representative chromatogram of M7 when separated on the UPLC-Q-TOF-MS/MS (**c**).

**Figure 8:**
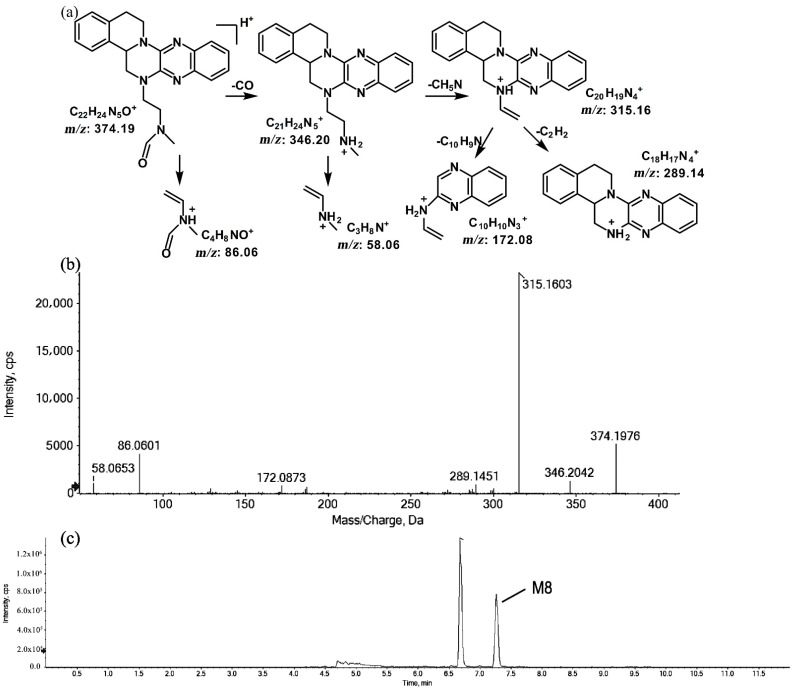
Suggested MS/MS fragmentation patterns of M8 identified to be C_22_H_23_N_5_O with *m*/*z* 374.19 (**a**), MS/MS spectra of M8 eluted at a retention time of 7.2 min (**b**), and the representative chromatogram of M8 when separated on the UPLC-Q-TOF-MS/MS (**c**).

The metabolites of *m*/*z* 374.19 were eluted at retention times of 6.6 (M7) and 7.2 min (M8), respectively. M7 and M8 were structural isomers with the same protonated molecular ions at *m*/*z* 374.19 and the same characteristic MS/MS product ions at *m*/*z* 315.16, *m*/*z* 289.14, and *m*/*z* 86.06. The molecular ions were 14 Da more than the prototype, while the characteristic product ions of the benzene rings were identical to those of the prototype. The results indicate that these metabolites should have resulted from oxidation on the side chain of the prototype. The characteristic product ions of M8 included *m*/*z* 346.20 and *m*/*z* 58.06. The fragmentation patterns of M7 are shown in Figure 7, while the fragmentation patterns of M8 are illustrated in Figure 8.

#### 2.1.4. Metabolites of *m*/*z* 390.19

**Figure 9:**
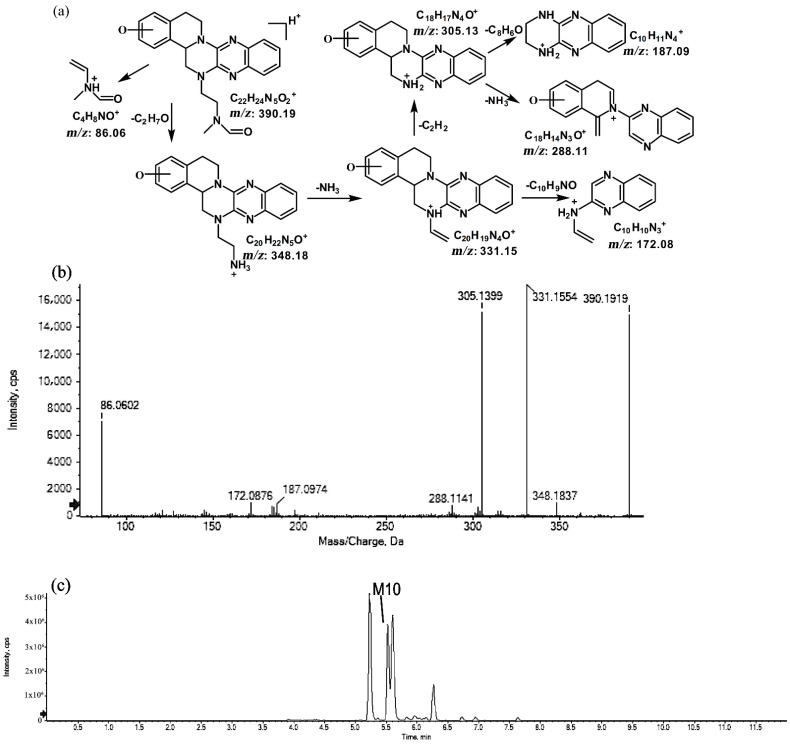
Suggested MS/MS fragmentation patterns of M10 identified to be C_22_H_23_N_5_O_2_ with *m*/*z* 390.19 (**a**), MS/MS spectra of M10 eluted at a retention time of 5.5 min (**b**), and the representative chromatogram of M10 when separated on the UPLC-Q-TOF-MS/MS (**c**).

**Figure 10:**
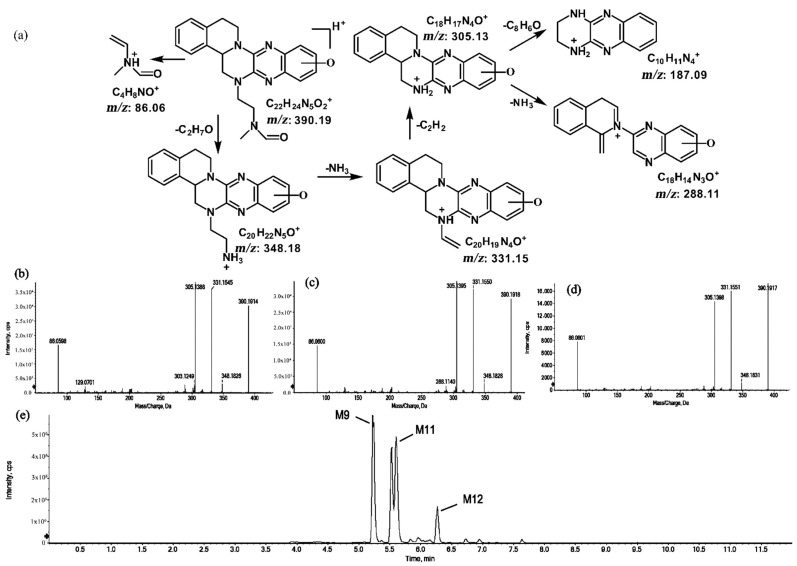
Suggested MS/MS fragmentation patterns of the group of structural isomers (M9, M11, and M12) identified to be C_22_H_23_N_5_O_2_ with *m*/*z* 390.19 (**a**), MS/MS spectra of M9 eluted at a retention time of 5.2 min (**b**), MS/MS spectra of M11 eluted at a retention time of 5.6 min (**c**), MS/MS spectra of M12 eluted at a retention time of 6.2 min (**d**), and the representative chromatograms of M9, M11, and M12 when separated on the UPLC-Q-TOF-MS/MS (**e**).

The metabolites of *m*/*z* 390.19 were eluted at retention times of 5.2, 5.5, 5.6, and 6.2 min. These metabolites (M9, M10, M11, and M12) were structural isomers with the same protonated molecular ions at *m*/*z* 390.19 and characteristic MS/MS product ions at *m*/*z* 348.18 and *m*/*z* 331.15. These isomers were identified to be C_22_H_23_N_5_O_2_, and the biotransformation pathway was proposed to be hydroxylation on ring A or ring E together with oxidation at the side chain. The product ion at *m*/*z* 172.08 indicates that the hydroxylation of M10 is on ring A. However, information about the specific site of the hydroxyl group on ring A could not be obtained from its MS/MS profile. The fragmentation patterns, representative spectra, and chromatogram of M10 are shown in Figure 9. By contrast, the hydroxylation of M9, M11, and M12 were on ring E. Similarly, information about the specific sites of M9, M11, and M12 on ring E could not be obtained from their MS/MS profiles. The fragmentation patterns, representative spectra, and chromatograms of M9, M11, and M12 are shown in Figure 10.

#### 2.1.5. Metabolites of *m*/*z* 392.20

**Figure 11:**
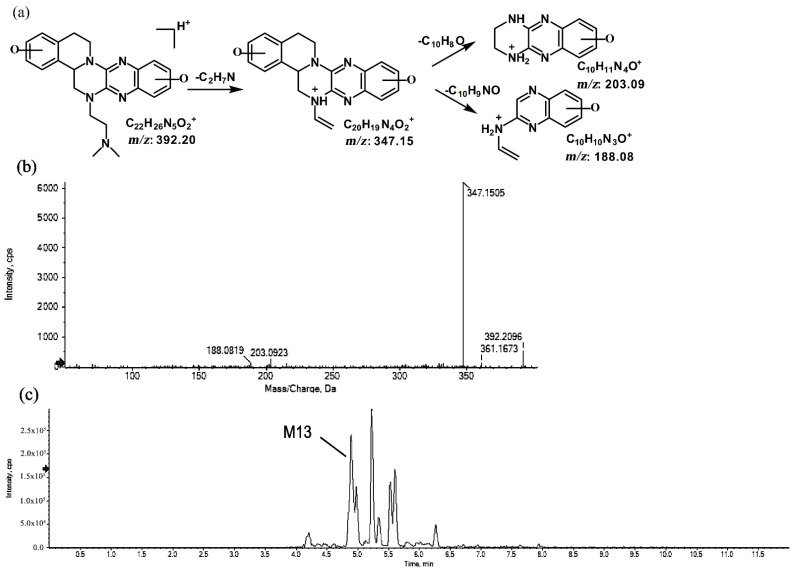
Suggested MS/MS fragmentation patterns of M13 identified to be C_22_H_25_N_5_O_2_ with *m*/*z* 392.20 (**a**), MS/MS spectra of M13 eluted at a retention time of 4.8 min (**b**), and the representative chromatogram of M13 when separated on the UPLC-Q-TOF-MS/MS (**c**).

The metabolite of *m*/*z* 392.20 was eluted at a retention time of 4.8 min (M13). M13, with the protonated molecular ion at *m*/*z* 392.20, and the characteristic MS/MS product ion at *m*/*z* 347.15 were both 32 Da greater than that of the prototype. Based on this result, the metabolic pathway of M13 was suggested to be di-oxidation. The proposed fragmentation patterns of M13 and its representative spectra and chromatogram are shown in Figure 11.

### 2.2. In Vivo Metabolites

A total of thirteen in vivo phase I metabolites of TNBG-5602 in rats after a single intravenous administration were found in the study. Five of these metabolites could be detected in both urine and feces, while the other eight metabolites could be detected only in the feces. The biotransformation pathways included mono-oxidation, demethylation, and hydroxylation together with oxidation and di-oxidation. The main in vivo metabolic sites included the amino group on the side chain along with ring A and ring E in the rigid coplanar ring structure of TNBG. The observed metabolites are shown in Table 2. In the table, the metabolites are differentiated by their molecular ions [M + H]^+^ and relative retention time, as resolved via the LC-MS/MS testing.

Phase II metabolites were rarely observed in this study, and further studies will be conducted to confirm this finding.

### 2.3. In Vitro Metabolites

The in vitro enzymatic metabolic pathways of TNBG-5602 were successfully confirmed. Six metabolites with different structures and types were detected in the incubation system of four human recombinant metabolizing enzymes. Since CYP2D6, CYP3A4, and CYP2C9 are the primary enzymes involved in the oxidation of exogenous substrates, while CYP4V2 has been reported to have the same substrates as PPARγ (the transcription factor of TNBG-5602) [16], these four isoforms of the enzyme were chosen to perform the in vitro metabolizing study. To elucidate the metabolic pathway, TNBG-5602 was incubated in the presence of recombinant CYP2D6, CYP3A4, CYP2C9, and CYP4V2. A complete list of oxygenated metabolites, following the incubation with the recombinant enzymes, is provided in Table 2.

For CYP2D6, it was found that six metabolites could be observed, i.e., M1–M4 with [M + H]^+^ at *m*/*z* 376.21, M5 with [M + H]^+^ at *m*/*z* 346.20, and M13 with [M + H]^+^ at *m*/*z* 392.20. For CYP3A4, it was found that only one metabolite can be obtained, i.e., M5, featuring [M + H]^+^ at *m*/*z* 346.20. Similarly, only M5 could be found in incubation samples with CYP2C9 or CYP4V2. M1–M4 and M13 could only be generated by incubating with CYP2D6, whereas M5 could be generated by all the enzymes in this study. As described above, M1–M4 and M13 could be the specific metabolites mediated by CYP2D6, while M5 is a common and non-enzyme-specific metabolite.

### 2.4. IC_50_ in Recombinant Human CYPs

**Figure 12:**
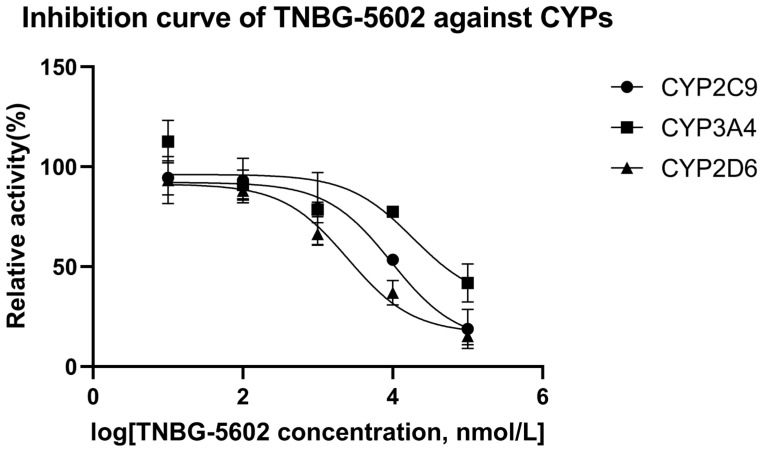
The inhibition curves for TNBG-5602 against cytochrome enzymes (mean ± SD, *n* = 3). TNBG-5602 (final concentrations of 0, 0.01, 0.1, 1.0, 10.0, and 100 μM) was incubated with recombinant cytochrome P450s (final concentration 0.025 μM) and the specific probe substates (final concentration 0.05 mg mL^−1^) with NADPH to initiate the metabolic reaction. The incubations were carried out at 37 °C for 30 min with a total volume of 200 μL. The CYP isoforms tested were 2D6 (dextromethorphan), 2C9 (tolbutamide), and 3A4 (testosterone). TNBG-5602 showed potent inhibition against CYP2D6, with an IC_50_ of 2.52 μM. TNBG-5602 showed moderate inhibition against CYP2C9, with an IC_50_ of 9.63 μM, as well as weak inhibition against CYP3A4, with an IC_50_ of 18.74 μM. The IC_50_ values were calculated using the GraphPad Prism statistical software (Version 8) by nonlinear regression analysis.

The results of the interaction between CYP450 enzymes and TNBG-5602 showed that TNBG-5602 had, competitively, an inhibitory effect on the metabolism of specific probe drugs of CYP2D6, and the IC_50_ results proved that TNBG-5602 had a potent inhibitory effect against CYP2D6, but it was moderate against CYP2C9 and weak against CYP3A4. Specific probe substrates were used to assess the interaction of TNBG-5602 with CYPs in this study. The inhibitory activity was reported as the IC_50_ calculated using the GraphPad Prism statistical software (version 8) by nonlinear regression analysis. Testosterone, tolbutamide, and dextromethorphan were chosen as the substrates for CYP3A4, CYP2C9, and CYP2D6 [17,18], respectively. TNBG-5602 showed potent inhibition against CYP2D6, with an IC_50_ of 2.52 μM. TNBG-5602 showed moderate inhibition against CYP2C9, with an IC_50_ of 9.63 μM, as well as weak inhibition against CYP3A4, with an IC_50_ of 18.74 μM. The inhibition curves for TNBG-5602 against the CYPs are shown in Figure 12.

### 2.5. Modeling

The interaction model between TNBG-5602 and metabolic enzymes was successfully established. The in vivo and in vitro results showed the essential role of CYP2D6 in the metabolism of TNBG-5602, as well as the strong interaction between them. However, other isoforms of the enzyme showed relatively moderate effects on the biotransformation of the compound. To further elucidate the specific mode of action between TNBG-5602 and these enzymes, a molecular-docking approach was used to conduct an in-depth study of the binding mode from a theoretical perspective.

**Figure 13:**
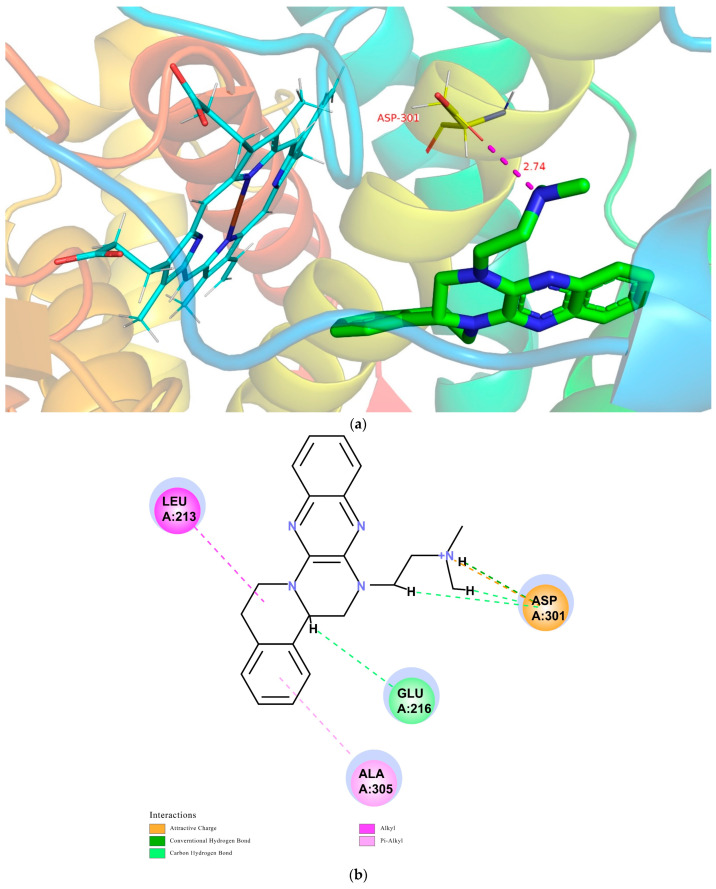
Conformations of compound TNBG-5602 docked into CYP2D6 (PDB code: 4XRY) with a CDOCKER INTERACTION ENERGY of -47.26 kcal mol^−1^: (**a**) 3D conformations showed that there was one NH group in TNBG-5602 that presented one hydrogen-bond interaction with ASP301 with a distance of 2.74 Å; (**b**) 2D conformations showed that attractive charge interactions, carbon–hydrogen-bond interactions, π-alkyl interactions, and alkyl interactions were observed. Molecular docking was performed using the Discovery Studio software package.

The CDOCKER module in DS2018 was utilized to study the probable interactions between TNBG-5602 and CYP2D6, CYP3A4, or CYP2C9. The related high-resolution crystal structures of CYP2D6 (PDB code: 4XRY), CYP3A4 (PDB code: 4D75), and CYP2C9 (PDB code: 5W0C) were selected for the molecular-docking analyses. All the water molecules were removed from the related enzymes before the docking calculation. In the protein structure, hydrogen atoms were added, and the CHARMm forcefield was applied for energy minimization. The binding site present in the enzyme was defined within the radii of 7.3, 6.4, and 7.4 Å. For convenience of comparison, TNBG-5602 was docked into the crystal structures of CYP2D6 (PDB code: 4XRY), CYP3A4 (PDB code: 4D75), and CYP2C9 (PDB code: 5W0C), and the top ten poses were examined on the basis of the CDOCKER INTERACTION ENERGY (protein–ligand interaction energy) to evaluate the nature and type of the interactions. Results suggest that TNBG-5602 docked well, with a CDOCKER INTERACTION ENERGY of -47.26 kcal mol^−1^ to 4XRY. As shown in Figure 13a, the result for TNBG-5602′s docking with 4XRY showed that there was one NH group in TNBG-5602 that presented one hydrogen-bond interaction with ASP301 with a distance of 2.74 Å. Attractive charge interactions, carbon–hydrogen-bond interactions, π-alkyl interactions, and alkyl interactions were also observed, as shown in Figure 13b. However, the results for TNBG-5602′s docking with CYP3A4 (PDB code: 4D75) and CYP2C9 (PDB code: 5W0C) showed that TNBG-5602 could not dock well with these two enzymes, as only a small number of weak interactions between them were observed.

Based on the theoretical calculation described above, the interaction between TNBG-5602 and CYP2D6 was much stronger than that with CYP3A4 or CY2C9. These results are also consistent with the in vivo and in vitro experimental results in this study. The modeling study results further confirm the primary role of CYP2D6 in the biotransformation of TNBG-5602. More importantly, these results indicate that TNBG-5602 can effectively bind to and interact with the target CYP2D6 through the alkyl side chain.

### 2.6. In Vivo Pharmacokinetics of TNBG-5602 in Rats

**Figure 14:**
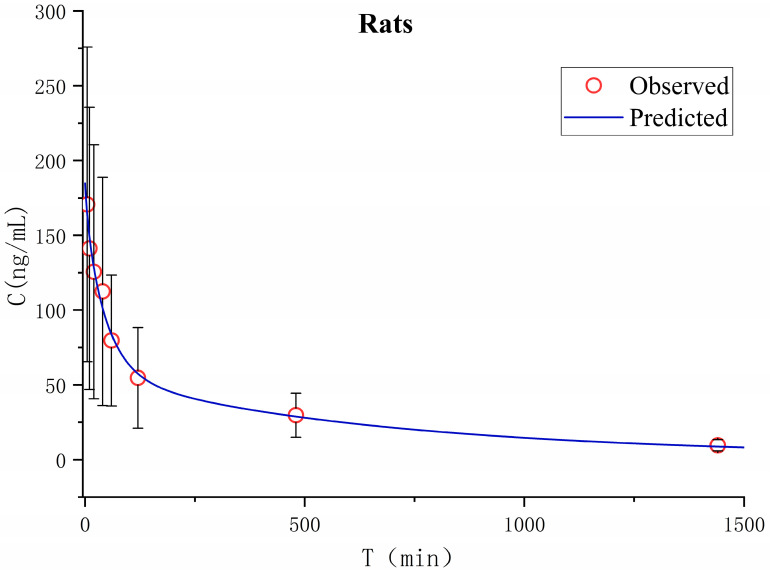
The mean plasma concentration–time curve for TNBG-5602 administrated intravenously at 4.8 mg kg^−1^ in rats (Mean ± SD, *n* = 5). Blood samples were collected before administration (0 min) and at 5, 10, 20, 40, 60, 120, 480, 600, and 1440 min after dosing. The plasma-concentration-time curve was analyzed on a two-compartment model using the Phoenix WinNonlin pharmacokinetic software package (Version 8.3, Certara).

Pharmacokinetics in rats after a single intravenous administration of TNBG-5602 was studied in vivo. The apparent volume of distribution in the peripheral compartment was 13.2 L, and the apparent clearance rate from the central compartment to the peripheral compartment was 0.028 ± 0.033 L min^−1^, indicating that the peripheral compartment, as a drug reservoir for TNBG-5602, played an important role in the distribution of TNBG-5602. The main pharmacokinetic parameters were calculated on the basis of the non-compartment and two-compartment analysis model using the Phoenix WinNonlin pharmacokinetic software package (version 8.3, Certara). The mean plasma concentration–time curves are shown in Figure 14.

The pharmacokinetic parameters were calculated using a non-compartment model, and the main pharmacokinetic parameters are listed in Table 3. After intravenous administration, TNBG-5602 could be rapidly distributed with a T_max_ of no more than 5 min. The apparent volumes of distribution were 20.2 L, and the total body clearance (CL) was 0.019 L min^−1^ in rats, with a half-life of 693 min upon intravenous administration at 4.8 mg kg^−1^.

Based on the concentration–time curves, the in vivo pharmacokinetic data for TNBG-5602 from rats were also fitted to a two-compartment model. The results were compared to those of the non-compartment model. As shown in Table 3, the clearance from the two-compartment model was 0.022 ± 0.009 L min^−1^, which did not show an obvious difference from that of the non-compartment model. The distribution volume of the peripheral compartment was 1.88-fold that of the central compartment, while the K_12_ was about 1.5-fold that of K_21_, indicating the important role of the peripheral chamber as a drug repository.

### 2.7. Validation Results of the Analytical Method for TNBG-5602 in Plasma

The HPLC-MS/MS method for pharmacokinetics was validated for parameters, including selectivity, linearity, precision, accuracy, extraction recovery, stability, and matrix effects.

**Figure 15:**
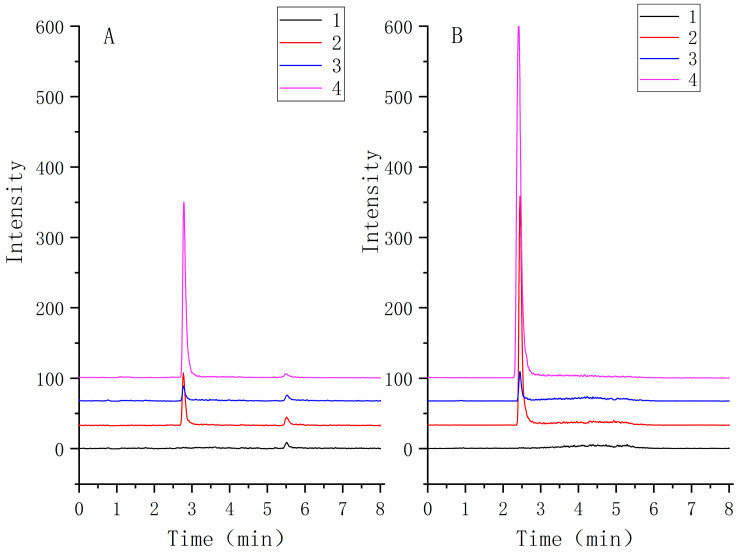
The representative MRM chromatograms of TNBG-5602 (**A**) and IS (**B**) for the quantitative determination of TNBG-5602. The separations were carried out by UPLC–MS/MS using an Agilent 1290 UPLC system coupled with a 6460 triple quadrupole mass spectrometer. The MRM transitions were 360.30 → 115.00 for TNBG-5602 and 336.20 → 320.10 for IS. From bottom to top: blank plasma (1); solvent spiked with TNBG-5602 and IS (2); blank plasma spiked with TNBG-5602 and IS (3); plasma sample from a TNBG-5602-treated rat (4).

The selectivity of the method was assessed by analyzing TNBG-5602 free plasma samples from six rats. The chromatograms of blank samples were compared to those of blank samples spiked with TNBG-5602 and internal standard (IS) and those of samples after the administration of TNBG-5602. The results suggested satisfactory selectivity of the method. The retention times of TNBG-5602 and IS were about 2.7 and 2.4 min, respectively. No impurities or endogenous substances were found to interfere with the quantitation of TNBG-5602 or IS. Representative mass spectra of the samples are shown in Figure 15.

The calibration curve was obtained by plotting the peak area ratio (y) of TNBG-5602 to IS versus the concentration (x) of TNBG-5602 standards. The linearity of the method was assessed via the standard curve. The slope, intercept, and coefficient of determinations were estimated using the least-squares linear regression method. The calibration curves of TNBG-5602 in rat plasma can be expressed as y = 0.0042x − 0.0046 in the range of 2.78–556.8 ng mL^−1^, with a correlation coefficient of r = 0.9998, showing satisfactory linearity. The LLOQ was 2.78 ng mL^−1^, with a signal-to-noise ratio of 10:1 and precision (RSD) within ±20%. These results demonstrate that the bioanalytical method offered sufficient sensitivity for the analysis of the analytes in plasma.

The intra-day and inter-day precision and accuracy of the method were evaluated by analyzing six replicates of QC samples at three different levels within the same day (intra-day) and over three consecutive days (inter-day), respectively. The precision is expressed as the RSD (%) of the QC samples, and the accuracy is expressed as the relative error (RE, %). According to the QC samples at the level of low (9.28 ng mL^−1^), medium (371 ng mL^−1^), and high (464 ng mL^−1^) concentration levels, the precision and accuracy for TNBG-5602 in plasma were evaluated. The intra-day and inter-day precision ranged from 2.09% to 6.11%, while the accuracy ranged from 5.86% to 13.58%. All the results, as shown in Table 4, satisfied the pre-specified acceptance criteria [20], indicating that this method was accurate and precise for the quantification of TNBG-5602 in bio-samples.

The matrix effect was evaluated by comparing the peak areas for the analytes spiked in post-extracted supernatant with those spiked in solvents at three different concentrations (*n* = 3). The recovery was obtained by comparing the peak areas for the analytes in QC samples spiked in blank plasma before extraction with the peak areas for analytes spiked in post-extracted supernatant at three different concentrations (*n* = 3). The extraction recoveries at the three QC levels ranged from 99.3 to 105.3%, with RSD (%) values of no more than 6.8% and the matrix effect ranging from 99.0 to 103.9%, with RSD (%) values of no greater than 6.4% (shown in Table 5). These results suggested that the extraction procedure was acceptable, as well as robust in terms of co-eluting substances on the ionization of the analytes.

Sample stability studies were performed, and the results indicated that samples were stable for at least 90 days when stored at −20 and −80 °C. The mean recoveries of QC samples (*n* = 3) after long-term storage and three freeze–thaw cycles (24 h interval for each) ranged from 102.6 to 105.8% at −20 °C and 102.6 to 108.6% at −80 °C, with SD values of no more than 10%. The stability results indicated that TNBG-5602 should be stable during the storage and analytical testing.

The peak areas of the blank samples injected after the highest linearity sample were less than 5% of the peak areas of the LLOQ samples, indicating that obvious carryover was not observed.

## 3. Discussion

**Figure 16:**
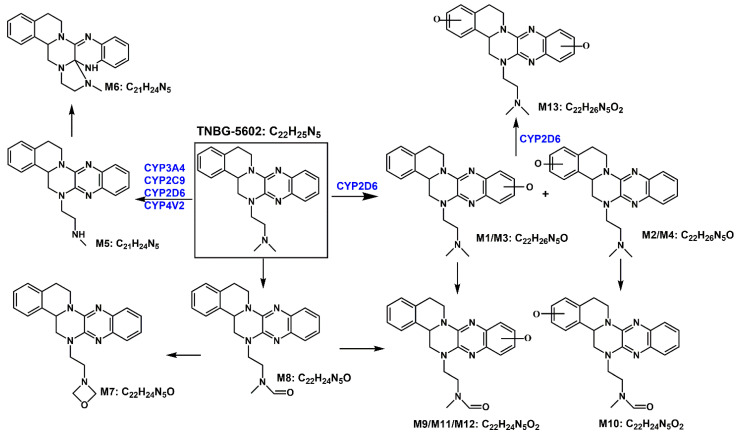
The proposed biotransformation pathways for TNBG-5602 based on the in vivo and in vitro metabolic study results.

Based on the in vivo and in vitro metabolic profile of TNBG-5602, the biotransformation pathways can be proposed as shown in Figure 16. The results of the in vivo metabolism demonstrated that TNBG-5602 could be effectively metabolized in vivo and that the metabolites could be excreted in the feces and urine. The in vitro study results showed that TNBG-5602 could be metabolized by CYP450s and that the main metabolites were consistent with the in vivo results. The main metabolic sites of TNBG-5602 could be the rigid coplanar ring (ring A and ring E) in its core structure and the side chain.

The metabolites detected in the feces and urine were not exactly the same, indicating that TNBG-5602 is metabolized by different enzyme isoforms in the processes of renal and liver excretion. There were five metabolites present in both the urine and feces, including M1, M2, M3, M4, and M5, while metabolites M6–M13 could only be found in the feces. M1–M5 had shorter retention times compared to the prototype when analyzed using reverse-phase chromatography, indicating that these metabolites have higher polarity and lower hydrophobicity than the prototype. Therefore, these metabolites could be easily excreted from the kidney. The retention times of M9–M11 and M13 were also shorter than those of the prototype, and they could only be detected in the feces. This suggests that M9, M11, and M13 could be secondary metabolites that mainly occur in the intestines.

The main metabolites in the urine were M1, M2, M3, M4, and M5, which are mainly metabolized through oxidation and N-demethylation. The major metabolites in the feces were M1, M2, M3, M5, M10, M11, and M12—that is, the main metabolic pathways featured the oxidation of the rigid coplanar ring, N-demethylation of the side chain, and oxidation of the rigid coplanar ring and the side chain. The minor metabolites in the feces were M4, M6, M7, M8, and M13; that is, the minor metabolic pathways were rearranged after N-demethylation, oxidation of the side chain, and di-oxidation.

Molecular docking revealed stronger interactions between TNBG-5602 and CYP2D6 compared to other isoforms of the enzyme, which was further confirmed by the IC_50_. These results suggest the essential role of CYP2D6 in the metabolism of TNBG-5602. TNBG-5602, as a potent inhibitor of CYP2D6, is likely to cause drug–drug interactions when used in a combination method for the treatment of cancer. Additionally, as the docking results showed, TNBG-5602 interacts with CYP2D6 mainly through the hydrogen bond formed between the NH on the side chain of TNBG-5602 and the ASP301 on CYP2D6. This finding provides a strong indication that modification of the side chain structure of the TNBG may significantly affect the metabolic behavior of this class of compounds.

The biliary excretion of, for example, glucuronic acid and glutathione conjugates was barely observed in this work. The possible reasons for this could be (1) the lack of a strong electrophilic group in the structure of TNBG-5602 that could be directly metabolized by the typical phase II transferases and (2) the potential suppression of glucuronosyltransferase and glutathione transferase. Further studies will be needed to confirm the hypothesis.

This is the first exploratory study on the PK properties of TNBG-5602 in rats. With intravenous administration, the dose was accurate and easy to control. Future PMDK studies using oral administration will be designed and conducted based on the results obtained in this work. In the in vivo pharmacokinetic assessment, TNBG-5602 showed a high volume of distribution, with the apparent volume of distribution being approximately 155-fold the total body water in rats. The in vivo clearance of TNBG-5602 was also high, with 200% liver blood flow in rats. Additionally, the half-life of TNBG-5602 was moderate, at nearly 12 h. Those results suggest that the recommended regimen for the drug candidate could be once a day to avoid accumulation. All these results indicate that TNBG-5602 could be distributed extensively to tissues with rapid elimination. Thus, TNBG-5602 showed a good metabolic profile and pharmacokinetic characteristics in vivo, which are important factors for the good developability of the drug candidate in future clinical trials.

Additionally, the in vivo clearance in rats exceeded the liver blood flow, as reported in the literature. This result indicates that TNBG-5602 could be eliminated by both hepatic and extrahepatic routes.

## 4. Materials and Methods

### 4.1. Reagents and Chemicals

TNBG-5602 (purity ≥ 99%) was synthesized in our laboratory at the College of Pharmacy, Chongqing Medical University (Chongqing, China). The berberine hydrochloride (purity ≥ 99%) used as an internal standard (IS) for the determination of TNBG-5602 was purchased from Dalian Meilun Biotechnology Co., Ltd. (Dalian, China). Carbamazepine (purity 100%), used as the internal standard for the determination of probe substrates, was purchased from China National Institutes for Food and Drug Control (Beijing, China).

Methanol and acetonitrile of HPLC grade were purchased from Honeywell B&J Co., Ltd. (Gyeonggi-do, South Korea). HPLC-grade formic acid was obtained from Fluka Industrial Co. (Shanghai, China). MILLIPORE Water (MILLIPORE S.A. Molsheim, France) was produced in house and used in the study. The solvents were passed through a 0.22-micron Nylon membrane before use.

A CYP450 Enzyme Inhibition Research Kit (IC_50_) for CYP3A4, a CYP450 Enzyme Inhibition Research Kit (IC_50_) for CYP2C9, and a CYP450 Enzyme Inhibition Research Kit (IC_50_) for CYP2D6 were purchased from IPHASE Innovative Reagents for Innovative Research. The constituents of the NADPH regeneration system in the kits were as follows: Solution A, including 26.1 mM of NADPH, 66 mM of glucose-6-phosphate, and 66 of mM magnesium chloride; Solution B, including 40U mL^−1^ of glucose-6-phosphate dehydrogenase and 5 mM of sodium citrate. The specific recombinant cytochrome P450 content in each kit was 100 pmol/mg protein, and the protein concentration was 10.0 mg/mL for each cytochrome P450 in these kits. CYP4V2 (human) recombinant protein (P01, 0.04 g L^−1^) was purchased from Abnova.

### 4.2. Animals

Male SD rats (weighing 190~210 g) were supplied by the Animal Centre of Chongqing Medical University (Chongqing, China). All the experiments in this study were approved by the Animal Research Ethics Committee of The Chongqing Medical University. All the rats were housed with free access to food and water under a standardized light/dark cycle condition (20~22 °C and 45~65% RH).

### 4.3. Instruments and Analytical Methods

#### 4.3.1. Qualitative LC-MS/MS Analysis of the Main Metabolites

An Exion LC^TM^ AC UPLC system (AB Sciex, Framingham, MA, USA) coupled with an X500R Q-TOF tandem mass spectrometer was used for qualitative analysis in this study. Chromatographic separation was carried out on an ACQUITY UPLC HSS T3 column (2.1 × 75 mm, 1.8 μm, Waters, Wexford, Ireland). The mobile phase consisted of Eluent A (0.1% aqueous formic acid) and Eluent B (acetonitrile: methanol, 80:20, *v*/*v*) at a flow rate of 0.3 mL min^−1^. The gradient elution program (%B ratio) was as follows: 5.0% at 0.0 min, kept at 5.0% from 0.0 to 1.0 min, increased to 95% from 1.0 to 8.0 min, kept at 95% from 8.0 to 10.0 min, decreased back to 5.0% at 10.1 min, and then held at 5.0% until 12.0 min. The column temperature was set to 35 °C. The injection volume was 5 μL. A full scan was performed over *m*/*z* 100–1000 for precursor ions in positive mode with a spray voltage of 5.5 kV, collision energy (CE) of 35 V, CE spread of 15 V, and accumulation time of 0.05 s. The Q-TOF scan for product ions was performed over *m*/*z* 100–1200 with CE 10 V and an accumulation time of 0.1 s.

#### 4.3.2. Quantitative LC-MS/MS Determination of Probe Substrates

The samples incubated in vitro for IC_50_ assessment were analyzed by UPLC–MS/MS (Agilent Technologies, Santa Clara, CA, USA) using an Agilent 1290 UPLC system coupled with a 6460 triple quadrupole mass spectrometer. The samples were prepared on a CSH C18 Column (3.0 × 75 mm, 1.7 μm). The LC-MS/MS instrument was the same as the system used to determine TNBG-5602. The mobile phase consisted of 0.1% formic acid, aqueous solution (Solvent A), and acetonitrile (Solvent B) at a flow rate of 0.3 mL min^−1^. The gradient elution program (%B) was as follows: 40% from 0.0 min to 1.0 min, increased to 65% for 0.01 min, and held constant for 1.01–3.0 min, followed by a decrease to 40% over 0.01 min while maintaining the composition over the total run time of 4.0 min. The column temperature was set at 30 °C. The injection volume was 1 μL. Detection was performed under the multiple reaction monitoring (MRM) mode with the positive-ion electrospray-ionization (ESI) mode. The MRM transitions were 271.10 → 91.00 for tolbutamide, 272.20 → 147.10 for dextromethorphan, 289.20 → 109.10 for testosterone, and 237.10→194.00 for carbamazepine.

A carbamazepine stock solution was prepared at 240 μg mL^−1^ in 50% acetonitrile aqueous solution and diluted to 1.042 μg mL^−1^ to produce the IS working solution. The working solutions of tolbutamide, dextromethorphan, and testosterone were all 10 mg mL^−1^. The working solutions of the three probe substrates were diluted to concentrations of 31.25, 25.00, 18.75, 12.50, and 6.25 μg mL^−1^ to prepare the calibration solutions. The calibration standards were prepared by spiking 20 μL of the calibration solutions at each concentration with 480 μL of IS working solution. The calibration curves were established by plotting the peak area ratio (y) of the probe substrate corresponding to IS versus the concentration (x) of the probe substrate in the calibration standards.

#### 4.3.3. Quantitative LC-MS/MS Determination of TNBG-5602

Plasma samples were analyzed by a UPLC–MS/MS method using an Agilent 1290 UPLC system coupled with a 6460 triple quadrupole mass spectrometer. Sample separation was carried out on a CSH C18 Column (3.0 × 75 mm, 1.7 μm). The mobile phase consisted of Eluent A (0.1% aqueous formic acid) and Eluent B (acetonitrile: methanol, 80:20, *v*/*v*) at a flow rate of 0.4 mL min^−1^. The gradient elution program (%B ratio) was as follows: 5.0% at 0.0 min, a linear gradient increase to 95% for 0.0~3.0 min, kept at 95% for 3.0~4.0 min, decreased back to 5.0% for 4.0~5.0 min, and then held at 5.0% until 8.0 min. The column temperature was set to 35 °C. The injection volume was 5 μL. The optimized spectrometric parameters were set as follows: a capillary voltage of 4 kV, gas temperature of 300 °C, gas flow rate of 5 L min^−1^, nebulizer of 45 Psi, sheath gas temperature of 250 °C, and sheath gas flow rate of 10 L min^−1^. The UPLC-MS/MS data were acquired and processed using the Mass Hunter MS Optimizer software (version B.07.00). Detection was performed under multiple reaction monitoring (MRM) mode with the positive-ion electrospray-ionization (ESI) mode. The MRM transitions were 360.30→115.00 for TNBG-5602 and 336.20→320.10 for berberine.

TNBG-5602 stock solution was prepared at 50 μg mL^−1^ in 0.1% aqueous formic acid. The TNBG-5602 stock solution was diluted with acetonitrile to obtain a series of working standard solutions for the calibration curve and QC samples. Berberine hydrochloride, the internal standard (IS), was dissolved in 0.1% aqueous formic acid to make the IS stock solution at 50 μg mL^−1^. The IS stock solution was then diluted with 0.1% aqueous formic acid to 50 ng mL^−1^ (IS working solution).

The calibration standards were prepared by spiking the appropriate amount of working standard solutions and 50 μL of IS working solution into 50 μL of rat blank plasma. The final concentrations of the calibration standards were 2.78, 4.64, 9.28, 46.40, 92.80, 185.6, and 556.8 ng mL^−1^. Calibration curves were established with these series of standards by plotting the peak area ratio (y) of TNBG-5602 to IS versus the concentration (x) of TNBG-5602 in the calibration standards. Quality-control samples were prepared at low (9.28 ng mL^−1^), medium (371 ng mL^−1^), and high (464 ng mL^−1^) concentrations.

### 4.4. Data Analysis

For the structural identification of metabolites, the UPLC–Q-TOF-MS/MS data were acquired and processed via SCIEX OS (Version 1.7, AB Sciex, Redwood City, CA, USA). The raw data files were imported into SCIEX OS 2.1 to search for and identify the metabolites of TNBG-5602. Identification of the chemical structure included two steps. The first step was automatic prediction, while the second step was manual validation. In Step 1, the candidate metabolites were predicted by the software based on the typical metabolic reactions of the templates. In Step 2, an ion chromatogram was extracted to screen candidate metabolites with mass tolerance within 5 ppm, using blank samples for the subtraction of the background. Then, the MS/MS fragment ions were extracted for the structural confirmation of candidate metabolites.

For IC_50_ assessment, the inhibition curves were established by nonlinear regression analysis plotting the relative activity (y) versus the logarithm of the TNBG-5602 concentration (x). The inhibition curve and the half-maximal inhibitory concentration of TNBG-5602 against each CYP isoform were analyzed using the GraphPad Prism statistical software (version 8). The equation used to calculate the IC_50_ values was Y = Minimal Inhibition + (Maximal Inhibition − Minimal Inhibition)/(1 + 10^(X-LogIC_50_)). The relative activity of each CYP isoform was calculated as follows:

(1) $$relative\;activity\;\%=\frac{C_{probe\;substrate\;added}-C_{n,probe\;substrate\;determined}}{C_{probe\;substrate\;added}-C_{0,probe\;substrate\;determined}}.$$

where $C_{0,probe\;substrate\;determined}$ is the probe substrate concentration determined in the blank groups (0 μM TNBG-5602) and $C_{n,probe\;substrate\;determined}$ is the probe substrate concentration determined in the TNBG-5602 groups at 0.01, 0.1, 1.0, 10.0, and 100 μM.

For pharmacokinetic studies of TNBG-5602 in rats, the main pharmacokinetic parameters were calculated on a non-compartment and two-compartment analysis model using the Phoenix WinNonlin pharmacokinetic software package (version 8.3, Certara).

### 4.5. In Vivo Studies in Rats

#### 4.5.1. Drug Administration

TNBG-5602 was dissolved in ultra-pure water to prepare the dosing solution at a concentration of 2.0 mg mL^−1^.

For in vivo metabolite studies, six rats were housed in separate metabolic cages, and each was administrated a dose of 4.8 mg kg^−1^ body weight via the caudal vein. The urine and feces samples were collected before administration (0 h) and at 8, 12, and 24 h after intravenous injection.

For the pharmacokinetic analyses, five rats were administered at a dose of 4.8 mg kg^−1^ body weight via the caudal vein. Blood samples (0.3 mL each) were collected via the orbital route into a 1.5 mL heparinized tube before administration (0 min) and at 5, 10, 20, 40, 60, 120, 480, 600, and 1440 min after intravenous injection.

#### 4.5.2. Treatment of Excretion Samples

Both urine and feces samples were collected and analyzed for in vivo metabolites.

The urine samples were vortex mixed with acetonitrile at a ratio of 1:3 (*v*/*v*) for 15 min. The mixture was then centrifuged at 15,000 rpm for 10 min, and the supernatant was dried using a gentle flow of nitrogen. The residue was dissolved in acetonitrile and vortex mixed again. The supernatant was dried again and re-dissolved in 0.1% aqueous formic acid: acetonitrile (50:50, *v*/*v*). The treated sample solutions were stored at −20 °C until analysis. A 5 μL aliquot of the thawed sample solution was injected into the UPLC–Q-TOF-MS/MS device to analyze the metabolites.

The feces samples were homogenized with pure water at a ratio of 1:1 (*w*/*w*). The homogenate was vortex mixed with acetonitrile at a ratio of 1:3 (*w*/*v*) for 15 min. The mixture was then centrifuged at 15,000 rpm for 10 min, and the supernatant was dried using a gentle flow of nitrogen. The residue was dissolved with acetonitrile and vortex mixed again. The supernatant was dried again and re-dissolved in 0.1% aqueous formic acid: acetonitrile (50:50, *v*/*v*). The treated sample solutions were stored at −20 °C until analysis. A 5-μL aliquot of the thawed sample solution was injected into the UPLC–Q-TOF-MS/MS device to analyze the metabolites.

#### 4.5.3. Treatment of Blood Samples

The whole blood samples were immediately centrifuged at 13,000 rpm for 5 min upon collection, and the supernatants were stored at −80 °C until analysis.

Obtained plasma (50 μL) was thawed and spiked with 50 μL of the IS working solution (50 ng mL^−1^). The mixture was deproteinized and extracted using 1 mL of acetonitrile by using a vortex for 2 min. After centrifugation at 12,000 rpm for 5 min, 5 μL of the supernatant was injected into the UPLC–MS/MS system to analyze TNBG-5602.

### 4.6. In Vitro Studies Using Recombinant CYPs

#### 4.6.1. Incubation with Recombinant CYPs

The stock solution of TNBG-5602 (5 mg L^−1^) was prepared using 0.1% aqueous formic acid. Then, the stock solution was diluted with PBS buffer to make the working solution of TNBG-5602 (1 mg mL^−1^). The working solution of TNBG-5602 was incubated with CYP2D6, CYP3A4, CYP2C9, and CYP4V2 recombinant cytochrome enzymes, respectively [14,21,22]. The total incubation volume for each reaction was 200 μL, containing TNBG-5602 (final concentration: 5 μg mL^−1^), cytochrome P450s (final concentration: 0.025 μM), 3.3 mM MgCl_2_, 1.3 mM NADPH, 3.3 mM glucose-6-phosphate, 0.4 U mL^−1^ glucose-6-phosphate dehydrogenase, and 0.05 mM sodium citrate. The cytochrome P450s were thawed on an ice bath and pre-incubated at 37 °C for 5 min. The mixtures were pre-incubated at 37 °C for 5 min before adding NADPH to initiate the reaction. NADPH was pre-incubated at 37 °C for 5 min and then added into the incubation system to initiate the reaction. The incubation periods were 60 min, and the reactions were quenched by adding 200 μL of iced acetonitrile. The samples were centrifuged (12,000× *g* rpm, 4 °C) for 10 min, and the supernatant was analyzed via the qualitative LC-MS/MS method. Blank samples in the absence of TNBG-5602 were incubated and followed by the same treatment.

#### 4.6.2. IC_50_ Assessment Using Recombinant Human CYPs

The stock solution of TNBG-5602 (200 mM) was prepared using 0.1% aqueous formic acid, and then diluted with PBS buffer to make working solutions at concentrations of 20, 2.0, 0.2, 0.02, and 0.002 mM. TNBG-5602 working solutions were incubated separately with recombinant cytochrome P450s and the specific probe substrates to measure the interactions between TNBG-5602 and CYP isoforms [23,24]. The CYP isoforms tested were 2D6 (dextromethorphan), 2C9 (tolbutamide), and 3A4 (testosterone) [25]. The total incubation volume for each reaction was 200 μL, containing TNBG-5602 (final concentrations of 0, 0.01, 0.1, 1.0, 10.0, and 100 μM), cytochrome P450s (final concentration: 0.025 μM), 3.3 mM MgCl_2_, 1.3 mM NADPH, 3.3 mM glucose-6-phosphate, 0.4 U mL^−1^ glucose-6-phosphate dehydrogenase, and 0.05 mM sodium citrate. The final concentration of the probe substrates in each reaction was 0.05 mg mL^−1^. The mixtures of TNBG-5602, probe substrates, and corresponding cytochrome P450s (final concentration: 0.025 μM) in a 0.1 M PBS buffer were pre-incubated at 37 °C for 5 min. NADPH was pre-incubated at 37 °C for 5 min and then added into the incubation system to initiate the reaction. The reaction mixtures were kept at 37 °C for 30 min, and 200 μL of iced acetonitrile was added to quench the reaction. The reaction mixtures were centrifuged (12,000× *g* rpm, 4 °C) for 10 min, and 20 μL of the supernatant was added to 480 μL of acetonitrile containing carbamazepine as the internal standard. The samples were vortexed for 2 min and then centrifuged for 10 min (12,000× *g* rpm). The final samples were analyzed for the probe substrates via the UPLC–MS/MS method described above.

### 4.7. Molecular Docking

Molecular docking was performed using the Discovery Studio software package. Specifically, it was carried out using the pharmacophore module called the “CDOCKER” protocol, which is an accurate molecular-docking strategy based on a CHARMm forcefield. The X-ray crystal structures of CYP2D6 (4XRY), CYP3A4 (4D75), and CYP2C9 (5W0C) complexed with related ligands were used as the reference receptor structures in the docking calculations and were downloaded from the Protein Data Bank of RSCB.

## 5. Conclusions

In conclusion, TNBG-5602, a novel isoquinoline derivative with targeted anticancer activities, exhibited favorable pharmacokinetic and metabolic characteristics in rats. The main metabolites of TNBG-5602 in rats were structurally identified, and the major metabolizing pathways were proposed to be mediated by oxidation and demethylation. This is the first report on the DMPK properties of TNBG-5602, an anticancer drug candidate. The results from the studies provide, with high confidence, a good basis for supporting TNBG-5602 clinical trials in human.

## Figures and Tables

**Table 1:** MS data obtained in positive-ion detection mode for TNBG-5602 and its metabolites along with their LC-MS/MS retention times and related *m*/*z* values. The analysis was carried out on an Exion LC^TM^ AC UPLC system (AB Sciex) coupled with an X500R Q-TOF tandem mass spectrometer. The UPLC-Q-TOF-MS/MS data were acquired and processed via SCIEX (Version 1.7).

Compound	Retention Time (min)	Relative Retention Time	*m*/*z* [M + H]^+^ Found	*m*/*z* [M + H]^+^ Calculated	Error (ppm)	Formula
Prototype	5.860	1.00	360.2180	360.2183	−0.8	C_22_H_25_N_5_
M1	4.900	0.84	376.2129	376.2132	−0.8	C_22_H_25_N_5_O
M2	5.099	0.87	376.2128	376.2132	−1.1	C_22_H_25_N_5_O
M3	5.262	0.90	376.2131	376.2132	−0.3	C_22_H_25_N_5_O
M4	5.499	0.94	376.2131	376.2132	−0.3	C_22_H_25_N_5_O
M5	5.739	0.98	346.2025	346.2026	−0.3	C_21_H_23_N_5_
M6	6.544	1.12	346.2026	346.2026	0.0	C_21_H_23_N_5_
M7	6.684	1.14	374.1972	374.1975	−0.8	C_22_H_23_N_5_O
M8	7.266	1.24	374.1976	374.1975	0.3	C_22_H_23_N_5_O
M9	5.233	0.89	390.1914	390.1925	−2.8	C_22_H_23_N_5_O_2_
M10	5.527	0.94	390.1921	390.1925	−1.0	C_22_H_23_N_5_O_2_
M11	5.604	0.96	390.1918	390.1925	−1.8	C_22_H_23_N_5_O_2_
M12	6.265	1.07	390.1917	390.1925	−2.1	C_22_H_23_N_5_O_2_
M13	4.895	0.84	392.2096	392.2081	3.8	C_22_H_25_N_5_O_2_

**Table 2:** In vivo and in vitro metabolites of TNBG-5602 analyzed by the UPLC-Q-TOF-MS/MS method.

Compound	Formula	RRT	Proposed Biotransformation	Urine	Feces	CYP2D6	CYP3A4	CYP2C9	CYP4V2
M1	C_22_H_25_N_5_O	0.84	P + O	×	×	×			
M2	C_22_H_25_N_5_O	0.87	P + O	×	×	×			
M3	C_22_H_25_N_5_O	0.90	P + O	×	×	×			
M4	C_22_H_25_N_5_O	0.94	P + O	×	×	×			
M5	C_21_H_23_N_5_	0.98	P-CH_2_	×	×	×	×	×	×
M6	C_21_H_23_N_5_	1.12	P-CH_2_		×				
M7	C_22_H_23_N_5_O	1.14	P + O-2H		×				
M8	C_22_H_23_N_5_O	1.24	P + O-2H		×				
M9	C_22_H_23_N_5_O_2_	0.89	P + 2O-2H		×				
M10	C_22_H_23_N_5_O_2_	0.94	P + 2O-2H		×				
M11	C_22_H_23_N_5_O_2_	0.96	P + 2O-2H		×				
M12	C_22_H_23_N_5_O_2_	1.07	P + 2O-2H		×				
M13	C_22_H_25_N_5_O_2_	0.84	P + 2O		×	×			

P: Prototype, TNBG-5602.

**Table 3:** The main pharmacokinetic parameters after a single intravenous administration of TNBG-5602 at 4.8 mg kg^−1^ in rats (*n* = 5). The parameters were calculated using the Phoenix WinNonlin pharmacokinetic software package (Version 8.3, Certara).

Non-Compartment Model			Two-Compartment Model		
Parameters	Mean	SD	Parameters	Mean	SD
AUC_(0–t)_ (μg L^−1^ min)	45513	21711	A (μg L^−1^)	127.2	74.3
AUC_(0–∞)_ (μg L^−1^ min)	56248	16507	B (μg L^−1^)	57.6	33.0
C_max_ (μg L^−1^)	170.7	105.2	K_10_ (1/min)	0.004	0.002
MRT_(0–t)_ (min)	427.6	105.0	K_12_ (1/min)	0.028	0.033
MRT_(0–∞)_ (min)	930.4	558.5	K_21_ (1/min)	0.019	0.026
t_1/2_ (min)	693.5	397.2	t_1/2α_ (min)	27.4	15.7
T_max_ (min)	5.0	0	t_1/2β_ (min)	710.9	425.8
CL_t_ (L/min)	0.019	0.005	CL (L/min)	0.022	0.009
V_d_ (L)	20.2	13.3	V_c_ (L)	6.985	3.545
			V_p_ (L)	13.2	9.4
Reported physiological liver blood flow for rats (200 g) (L/min) [19]				0.011	
Reported physiological total body water volume (200 g) (L) [19]				0.13	

K_10_: Apparent first-order constant of the rate of elimination from the central compartment. K_12_: Apparent first-order constant of the rate of intercompartmental transfer from the central compartment to the peripheral compartment. K_21_: Apparent first-order constant of the rate of intercompartmental transfer from the peripheral compartment to the central compartment. A: Zero-time intercept of the α distribution phase. B: Zero-time intercept of the elimination phase. V_d_: Apparent volume of distribution. V_c_: Apparent volume of the central compartment. V_p_: Apparent volume of the peripheral compartment.

**Table 4:** Intra-day and inter-day precision and accuracy results of the analytical method for TNBG-5602 in rat plasma by UPLC-MS/MS method.

Concentration Level	Precision (RSD, %)		Recovery (Mean ± SD %)	
	Intra-Day (*n* = 6)	Inter-Day (*n* = 18)	Intra-Day (*n* = 6)	Inter-Day (*n* = 18)
Low (9.28 ng mL^−1^)	5.44	4.20	112.8 ± 6.13	113.6 ± 4.77
Medium (371 ng mL^−1^)	2.09	3.84	105.9 ± 2.22	108.3 ± 4.16
High (464 ng mL^−1^)	2.91	6.11	106.3 ± 3.09	108.4 ± 6.62

**Table 5:** Mean extraction recoveries of the analytical method for TNBG-5602 in rat plasma (*n* = 3) by the UPLC-MS/MS method.

Concentration Level	Extraction Recovery		Matrix Effect	
	Mean (%)	RSD (%)	Mean (%)	RSD (%)
Low (9.28 ng mL^−1^)	105.3	6.8	103.9	6.4
Medium (371 ng mL^−1^)	99.3	3.6	99.0	3.2
High (464 ng mL^−1^)	100.8	5.4	103.0	4.3

## Data Availability

The data presented in this study are available on request from the corresponding author.

## Sample Availability

Samples of the compounds TNBG-5602 are available from the authors.
